# Impact of thyroid function abnormalities on reproductive hormones during menstrual cycle in premenopausal HIV infected females at NAUTH, Nnewi, Nigeria

**DOI:** 10.1371/journal.pone.0176361

**Published:** 2017-07-19

**Authors:** Nkiruka Rose Ukibe, Solomon Nwabueze Ukibe, Obiageli Fidelia Emelumadu, Chinedum Charles Onyenekwe, Joseph Eberendu Ahaneku, Anthony Osita Igwegbe, Ifeoma Nwamaka Monago, Amobi Linus Ilika

**Affiliations:** 1 Department of Medical Laboratory Science, College of Health Sciences, Nnamdi Azikiwe University, Nnewi Campus, Nnewi, Anambra State, Nigeria; 2 Department of Prosthesis and Orthotics, School of Health Technology, Federal University of Technology Owerri, Imo State, Nigeria; 3 Depatment of Community Medicine, College of Health Sciences, Nnamdi Azikiwe University, Nnewi, Anambra, State Nigeria; 4 Department of Chemical pathology, College of Health Sciences, Nnamdi Azikiwe University, Nnewi Campus, Nnewi, Anambra State, Nigeria; 5 Department of Obstetrics and Gynecology, College of Health Sciences, Nnamdi Azikiwe University, Nnewi Campus, Nnewi, Anambra State, Nigeria; 6 Department of Medical Services, Federal Polytechnic, Oko, Anambra State, Nigeria; Universite Clermont Auvergne, FRANCE

## Abstract

**Background:**

This was a prospective study designed to evaluate the impact of thyroid function abnormalities on reproductive hormones during menstrual cycle in HIV infected females at Nnamdi Azikiwe University Teaching Hospital Nnewi, South-East Nigeria.

**Methods:**

The study randomly recruited 35 Symptomatic HIV infected females and 35 Symptomatic HIV infected females on antiretroviral therapy (HAART) for not less than six weeks from an HIV clinic and 40 apparently heathy control females among the hospital staff of NAUTH Nnewi. They were all premenopausal females with regular menstrual cycle and aged between 15–45 years. Blood samples were collected at follicular and luteal phases of their menstrual cycle for assay of Thyroid indices (FT3, FT4 and TSH) and Reproductive indices (FSH, LH, Estrogen, Progesterone, Prolactin and Testosterone) using ELISA method.

**Results:**

The result showed significantly higher FSH and LH but significantly lower progesterone (prog) and estrogen (E2) in the test females compared to control females at both phases of menstrual cycle (P<0.05). There was significantly lower FT3 but significantly higher TSH value in Symptomatic HIV females (P<0.05). FSH, LH and TSH values were significantly lowered while prog and FT3 were significantly higher in Symptomatic HIV on ART compared to Symptomatic HIV females (P<0.05). FT3, FT4, Prog and E2 were inversely correlated while FSH and LH were positively correlated with duration of HIV infection in HIV females (P<0.05 respectively). There was a direct correlation between CD4+ count and FT3 while inverse correlation was found between CD4+ count and TSH levels (P<0.05).

**Discussion:**

The present study demonstrated hypothyroidism with a significant degree of primary hypogonadism in Symptomatic HIV infected females at both follicular and luteal phases of menstrual cycle which tends to normalize on treatments.

## Introduction

One of the dreaded complications of HIV/AIDS is its effects on the endocrine function of affected men and women thereby raising reproductive problems that may defy solutions. It has been reported that imbalances in sex hormones may be the most common endocrine disorders in HIV- positive people [1]. Hypogonadism- low testosterone in men and decreased levels of estrogen, progesterone, and/or testosterone in women- can lead to a myriad of problems including impaired sexual function and decreased fertility. Reduced sex hormone levels in women can cause menstrual irregularities such as ammenorrhoea, polymenorrhoea or oligomenorrhoea. Some authors have associated advanced HIV disease with one form of menstrual irregularity or the other [2–4]. Grinspoon *et al*. attributed the menstrual irregularities observed in women with advanced HIV/AIDS to metabolic imbalances and cachexia or weight loss [3]. All these menstrual problems are believed to have their origin in abnormality of sex hormones in these women.

The connection between thyroid hormone levels and the menstrual cycle is mainly mediated by thyrotropin-releasing hormone (TRH), which has a direct effect on the ovary and abnormal thyroid function can alter levels of sex hormone-binding globulin, prolactin, and gonadotropin-releasing hormone, contributing to menstrual dysfunction. For example, increased levels of TRH may raise prolactin levels, contributing to the amenorrhea associated with hypothyroidism [5].

In women in their reproductive age, thyroid autoimmunity is the most prevalent cause of thyroid dysfunction [6, 7].

Recent study has reported that thyroid receptors (TRs) are also present in human ovarian surface epithelium and act on ovarian follicles which show some slight localization in granulosa cells of ovarian follicles [8]. THs regulate a variety of biological processes including growth, cellular oxygen consumption, metabolism, embryonic development, tissue differentiation, and maturation [9]. The down-regulation of TRs in mammals has been shown to lower the fertility and decrease follicle number [10] and this may worsen in disease conditions. Hypothyroidism has been associated with the altered ovarian function, menstrual irregularities, subfertility, and higher (recurrent) miscarriage rates, suggesting that thyroid hormone affects female reproductive axis [11, 12].

Hypothyroidism causes an increase in the levels of thyroid releasing hormone (TRH) which in turn stimulates secretion of thyroid stimulating hormone (TSH) and prolactin (PRL) and PRL inhibits the synthesis and secretion of gonadotrophins.

The advent of highly active antiretroviral drugs (HAART) seem to reduce the frequency of menstrual irregularities in women with HIV disease [13], although reports of hypermenorrhoea have been documented [14] especially in women receiving protease inhibitors particularly retonavir (norvir).Studies have also shown increased prevalence of hypothyroidism in patients with HIV illness who have been placed on antiretroviral therapy [15, 16]. Hormonal changes in thyroid gland in HIV disease are believed to be due to release of cytokines especially interleukin-6 (IL-6) and tumor necrosis factor (TNF). Some researchers have reported a rise in T3 and T4 levels in the early stage of HIV infection and a fall in T3 level with the progression to AIDS [17, 18]. Hoffman and Brown reported increased prevalence of thyroid abnormalities in HIV-infected subjects [17].

Croxson *et al*. reported normal prolactin levels in HIV affected individuals with normal response to TRH stimulation [18]. However, Hutchinson *et al*. reported hyperprolactinaemia with galactorrhoea in four HIV-infected women who were on protease inhibitors [19]. The endocrine abnormality was attributed to the drug use directly or the indirect effect of protease inhibitor on cytochrome P450 which potentiates the Dopamine antagonist effect of other drugs [19].

Hence, the present study seeks to assess the impact of thyroid abnormality on the reproductive life of HIV infected females within their reproductive age.

## Materials and methods

**Subjects:** The study consist of 110 randomly selected premenopausal females aged between 15and 45years which includes: 35 Symptomatic HIV infected females aged (38.63 ±1.65 years) and 35 HIV infected females on HAART for not less than six months aged (37.11±13.24 years) recruited at HIV Clinic and 40 apparently healthy Control females aged (39.95±10.67 years) with regular menstrual cycle recruited among the hospital staff of Nnamdi Azikiwe University Teaching Hospital Nnewi, South-East, Nigeria. All the participants were screened for HIV and TB and were classified into the various groups using WHO and CDC criteria for HIV.

A well-structured questionnaire was administered to each participant to ascertain the history of their menstrual cycle, reproductive history and other biodata such as age, duration of HIV infection types of therapeutic regimens. The course of the disease was defined as the duration of HIV infection calculated from the first HIV positive test result [15] and the course of HAART was calculated from the day on which HAART was introduced.

### Blood sample collection

Six ml of blood sample was collected from each participant at follicular (7-13^th^ day) and at luteal (21-23rd day) phases of menstrual cycle. The blood sample was collected between 8 to 10am by venepuncture. Four ml was dispensed into dry plain bottles and allowed to clot, retracted and centrifuged. The serum was separated from the clot immediately and transferred into a well labeled container and stored frozen at -20^0^c until assayed for hormones (FSH, LH, prolactin, progesterone, estradiol, FT3, FT4 and TSH. The remaining two mls of blood was dispensed into EDTA bottles and was used immediately for malaria parasite screening HIV screening and confirmation.

### Ethical clearance and informed consent

The Ethics Committee of Nnamdi Azikiwe University Teaching Hospital Nnewi, Anambra state, Nigeria approved the study design. The participants were informed about the study design and only those who gave their consent were recruited for the study. The informed consent form was written and approved along with the ethical clearance obtained from the ethics committee board of NAUTH Nnewi. The consent form was issued with the questionnaire during recruitment which only those who agreed and volunteered to participate signed before their blood samples were collected. All the participants recruited were assured that information obtained from them would be treated with uttermost confidentiality and they had the full right not to participate in or withdraw from the study at any point they desired to. The ethics committee approval included that of individuals from 15years to 45years and the written informed consent for participant between 15 and 18 years was obtained from their parents/guardians.

### Exclusion and inclusion criteria

Participants with HIV Stage-1 (Asymptomatic HIV), HIV Stage –3 and 4 were excluded from the study. Only those adjudged as symptomatic-HIV (stage-2) were included in the study. Participants with malaria parasite infection as at the time of study were also excluded. Participants with extra pulmonary tuberculosis were excluded and subjects with known fertility problems before contracting HIV infections were also excluded. Women on any contraceptives were also excluded. Hence the female participants used were those with no prior fertility problems until the existence of HIV.

### Highly active antiretroviral drugs administration

Participants on highly active antiretroviral therapy were given either of the following regimens: generic fixed-dose combination of (1) zidovudine (ZDV) 300 mg twice daily orally + lamivudine (3TC) 150 mg twice daily orally + nevirapine (NVP) 200 mg twice daily orally. (Zidovudine and Lamivudine are nucleoside reverse transcriptase inhibitors while niveripine is a non-nucleoside reverse transcriptase inhibitor (NNRTI) or (2) Stavudine (D4T) 30 mg twice daily orally + Lamivudine (3TC) 150 mg twice daily + Nevirapine (NVP) 200 mg twice orally. (Stavudine is also a nucleoside reverse transcriptase inhibitor).

### Methods

Antibodies to HIV-1 and HIV-2 in Human Plasma were detected using Abbott Deterimine system, Immunoassay method [(Trinity Biotech UniGold Assay Kit (Trinity Biotech PLC, Ireland)] and imunochromatographic method [(HIV 1 and 2 STAT-PAK Assay kit (Chembio diagnostic system, INC New York, USA)] respectively.

Determination of Progesterone, Estradiol Prolactin, Free Triiodothyronine, Free Thyroxine and Thyrotropin (FT3, FT4 and TSH) were done using Enzyme Linked Immunosorbent Assay (ELISA) kits (Glory Science Laboratory USA) CD4+ t-cell count was done by the Becton Dickinson FACS flow cytometer. Plasma HIV viral load (HIV RNA) was done using reverse transcriptase- polymerase chain reaction assay (Cobas Amplicor HIV-1 Monitor Test, version 1.5, Ultrasensitive specimen preparation, Roche Diagnostic System Inc, Branchburg, NJ USA). The lower limit of detection in plasma was 50 HIV-1 RNA copies/ml.

### Statistical analysis

The version 16 of SPSS package was used in statistical analysis. The variables were expressed as mean (±SD). The Student independent t-test and analysis of variance (ANOVA) and post-hoc (LSD) were used to assess significant mean differences. Percentage and χ^2^ were used for categorical measures. Graph Pad Prism version 5.03 was used for graph presentations. The Pearson correlation coefficient was used to assess the association between numerical / continuous variable and spearman correlation coefficient was used to assess the association between discrete variables. All comparisons were two sided and the level of significance was considered at P<0.05. Univariate and multivariate logistic regression analyses were done and probability for stepwise was 0.1 to entry and 0.15 to removal.

## Results

### Demographic and clinical characteristics of HIV infected subjects

The demographic and clinical characteristics shows that the mean (±SD) age (years) of symptomatic HIV females (38.63 ± 10.65) and symptomatic HIV infected females (37.11 ± 13.24) on HAART were not significantly different compared to control group (39.95 ± 10.67) (P>0.05). The mean BMI (kg/m2) was significantly higher in symptomatic HIV infected females (23.5 ± 1.67) and symptomatic HIV infected females on HAART (24.0 ± 3.80) compared with control (20.83 ±0.81) subjects (P<0.05). However, duration of HIV infection in symptomatic HIV infected females (8.19 ± 4.01) years was significantly shorter compared with symptomatic HIV infected females on HAART (17.26 ± 6.84 years) (P<0.05). The mean CD4+ T- cell count (cells/μL) in symptomatic HIV infected females (287 ± 178) and symptomatic HIV infected females on HAART (443 ± 216) was significantly lower when compared with control (634 ± 256) (P<0.05 respectively). Also, CD4+ count in symptomatic HIV females was significantly lower compared with the counterpart on HAART (P<0.05). Similarly, the viral load in symptomatic HIV female was significantly higher [10E (6.34 ±2.45)] compared to the counterpart on HAART [10E (1.08 ± 1.14)] (P<0.05). Contrastingly, nadir CD4+ T-cells (cells/μL) was not significantly different between symptomatic HIV infected females (212 ± 109) and their corresponding females on HAART (296 ± 98) (P>0.05). Menstrual irregularities percentage was higher in symptomatic HIV infected females 18(40%) as compared to symptomatic HIV infected females on HAART 28(25.7%) while 34(34.3%) of HIV females have regular menstrual cycles (P<0.05). There was no significant difference observed between symptomatic HIV females who were receiving zidovudine (NRTI), lamivudine (NRTI) and nevirapine (NNRTI) and those who were receiving Stavudine (NRTI), Lamivudine (NRTI), and Nevirapine (NNRTI) (P>0.05) (See Table 1).

**Table 1:** Demographic and clinical characteristics of HIV infection.

Parameters	Symptomatic HIV infected Females (n = 35)	Symptomatic HIV infected Females on ART (n = 35)	Control (n = 40)	t-test	P-value
Age (years) (Mean ±SD)	38.63 ±10.65	37.11 ± 13.24	39.95 ± 10.67	1.181	0.681
BMI (Kg/m^2^)	23.50 ± 1.67	24.00 ± 3.80	23.83 ± 0.81	5.206	0.019
Duration of HIV infection (years)	8.19 ± 4.01	17.26 ± 6.84	n/a	4.894	0.001
CD4 T-cell count cells/μL	247 ± 178	443 ± 216	634 ± 256	6.878	0.001
Log Viral load VL	6.34 ± 2.45	1.08 ± 1.14	n/a	5.102	0.001
Nadir CD4+ T-cell count cells/μL	184 ± 79	236 ± 138	n/a	4.997	0.012
Menstrual Irregularity	28 (40%)	18(25.7%)	-	4.593	0.006
Nature of HAART					
NRTI	n/a	18(51.4%)	n/a	1.031	0.312
NNRTIs	n/a	17(48.6%)	n/a		

BMI = Basal metabolic index, HAART = highly active antiretroviral therapy, NRTI = Nucleoside Reverse Transcriptase Inhibitor, NNRTIs = Non-Nucleoside Reverse Transcriptase Inhibitors

n/a = not available, Level of significance was considered at P<0.05

FT3, FT4, Prog and E2 were inversely correlated while FSH and LH were positively correlated with duration of HIV infection in HIV females (P<0.05 respectively) (Table 2). There was a direct correlation between CD4+ count and FT3 while inverse correlation was found between CD4+ count and TSH levels (P<0.05). (See Table 3). Univariate and multivariate logistic regressions were used to predict effect of thyroid and reproductive dysfunctions on HIV infected women irrespective of the phases of menstrual cycle after adjusting for possible confounding variables age, BMI, viral load, CD4 count and HAART. It was observed that in univariate analyses, the duration of HIV infection with odd ratio (OR) (1.101) and 95% confidence interval (CI) (1.011–1.080) (P<0.05), nadir CD4+ count with OR (1.989) and CI (1.374–2.100) (P<0.05), E2 with OR (-0.154) and CI (-0.138, -0.162) (P<0.05) were significantly associated with thyroid and reproductive dysfunction. The multivariate analysis also showed similar results with the univariate analysis for duration of HIV, CD4+ nadir T cell count and E2 with OR and CI of 1.091(1.137–1.748) (P<0.05), 1.898 (1.393–2.104) (P<0.05), -0.156(-0.139,- 0.168) (P<0.05) (See Table 4).

**Table 2:** Correlation of thyroid function tests and sex hormones levels with HIV duration in HIV females.

HIV DURATION		
Parameters	r-value	P- value
FT3 (ng/ml)	-0.2301	0.041
FT4 (μg/dl)	-0.2443	0.044
FSH (mIU/ml)	0.2294	0.005
LH (mIU/ml)	0. 4451	0.019
E2 (pg/ml)	-0.2705	0.012
Prog (ng/ml)	-0.2248	0.008

FT3 = Free triiodothyronine, FT4 = free thyroxine, FSH = Follicle stimulating hormones, LH = luteinizing hormones, E2 = Estrogen, Prog = Progesterone

**Table 3:** Correlation between thyroid function tests and CD4+ count.

CD4+ count cells/μL		
Parameters	r-value	P- value
FT3 (ng/ml)	0.4233	0.002
TSH (μIU/ml)	-0.2911	0.018

TSH = Thyroid stimulating hormones

**Table 4:** Univariate and multivariate logistic regression models.

		95% CI		
	Odd ratio			P-Value
		Lower	Upper	
Univariate logistic regression				
Duration of HIV infection	1.101	1.011	1.080	0.012
Nadir CD4+ count cells/μL	1.989	1.374	2.100	0.044
LH(mIU/ml)	-0.073	-0.066	-0.090	0.520
FSH (mIU/ml)	-0.084	-0.054	-0.079	0.328
E2 (pg/ml)	-0.154	-0.138	-0.162	0.030
Multivariate logistics regression				
Duration of HIV infection	1.091	1.137	1.748	0.009
Nadir CD4+ count cells/μL	1.898	1.393	2.104	0.023
LH(mIU/ml)	-0.078	-0.065	-0.095	0.538
FSH (mIU/ml)	-0.065	-0.041	-0.047	0.340
E2 (pg/ml)	-0.156	-0.139	-0.168	0.035

Univariate and Multivariate logistic regressions after adjusting for possible confounding variables age, BMI, viral load, CD4 count and HAART.

### Levels of anterior pituitary hormones {(FSH (mIU/ml), LH (mIU/ml) and prolactin (ng/ml)} at follicular and luteal phases of menstrual cycle

The mean (±SD) serum FSH, LH and Prolactin Levels in Symptomatic HIV females were not significantly different between follicular (19.4±10.9, 18.0±16.6, 19.3±5.5) and luteal (26.3±5.2, 14.0±4.2, 18.65±4.8) phases of menstrual cycle (P>0.05 respectively). Similarly, there was no significant difference in the mean LH and Prolactin concentrations between follicular (10.6±5.4, 18.0±3.5) and luteal (13.7±4.5, 18.0±3.5) phases of menstrual cycle in Symptomatic HIV female subjects on HAART (P>0.05) but the mean FSH value dropped significantly at luteal (13.5±2.5) phase compared with follicular (18.2±10.4) phase of menstrual cycle in Symptomatic HIV females on HAART (P<0.05). The mean serum FSH and LH values in Control females were significantly higher at follicular phase (8.7±3.4, 7.9±6.3) than at luteal phase (4.4±1.4, 4.4±1.9) of menstrual cycle (P<0.05 respectively). There was no significant difference in the mean prolactin value between follicular (18.3±4.4) and luteal (18.1±3.4) phases of menstrual cycle in Control female subjects (P>0.05).

The mean FSH and LH were significantly higher in Symptomatic HIV (19.4±10.9, 12.11±5.46) compared with the corresponding value in the Control female subjects (7.9±6.3) (P<0.05). FSH was significantly higher at follicular phase in Symptomatic HIV on HAART (18.2±10.4) compared with corresponding value in the Control female subjects (7.9±6.3) (P<0.05). The mean FSH and LH concentration at luteal phase of menstrual cycle were significantly higher in Symptomatic HIV (26.3±5.2, 12.60+5.34) and Symptomatic HIV on HAART (13.5±2.5, 11.9+6.2) compared with corresponding value in the Control female subjects (4.4±1.4) (P<0.05 in each case).

**Fig 1:**
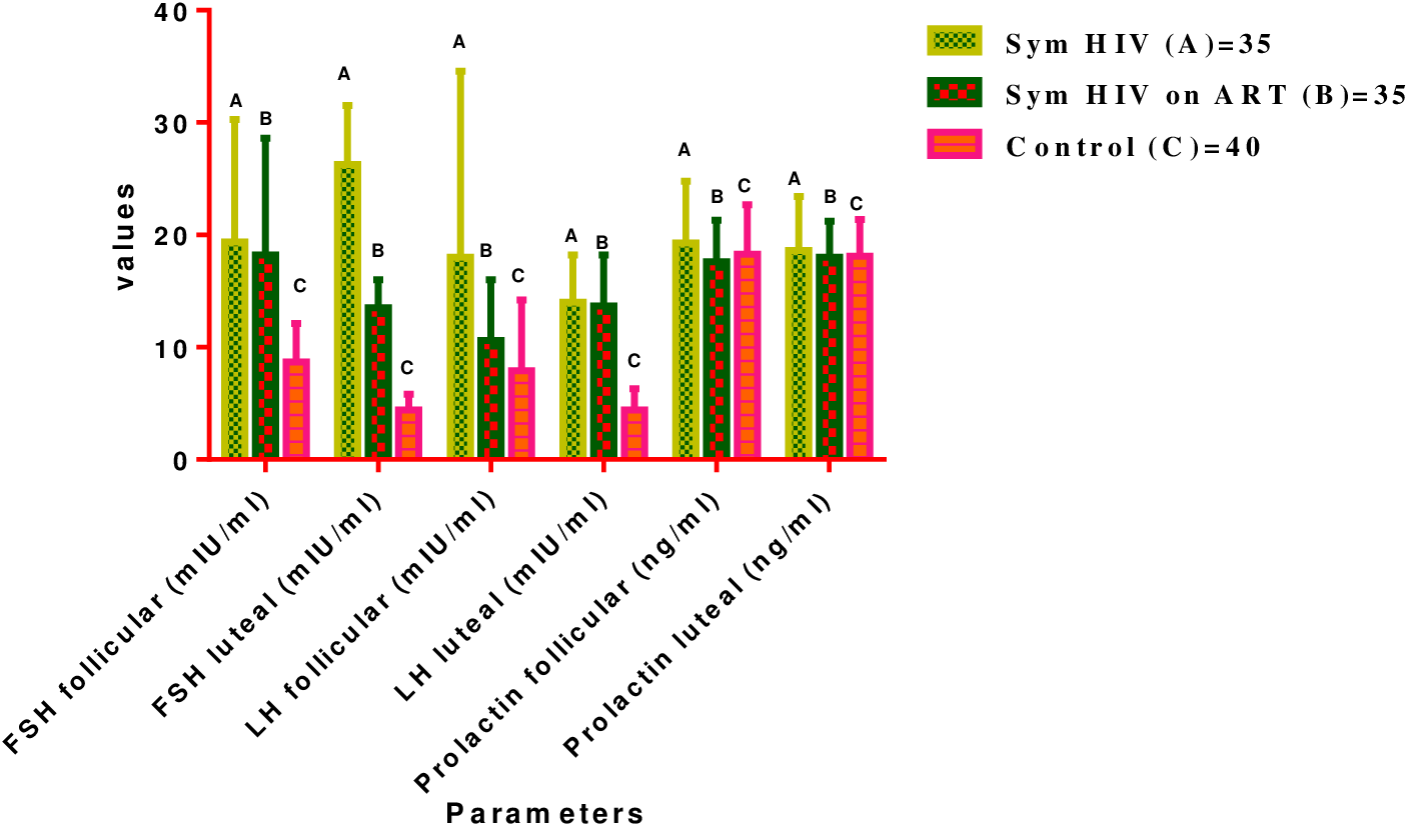
Comparison of mean (±SD) serum levels of FSH, LH and prolactin in test groups and control group at follicular and luteal phases of menstrual cycle. Key- FSH in all Test groups vs C = P<0.05 at both phases of menstrual cycle. FSH in B = P<0.05 between follicular and luteal phases of menstrual cycle LH in A vs C = P<0.05 at both phases. B vs C = P<0.05 at luteal phase. A vs B = P<0.05 at both phases of menstrual cycle.

The post hoc analysis showed significant drop in the mean LH value at follicular phase of menstrual cycle in Symptomatic HIV females on HAART (10.6±5.4) compared with the corresponding value in Symptomatic HIV female subjects (18.0±16.6) (P<0.05) (See Fig 1).

### Levels of steroid hormone {(progesterone (ng/ml and estradiol (pg/ml)} at follicular and luteal phases of menstrual cycle

The mean (±SD) serum progesterone and estradiol concentrations in Symptomatic HIV females were not significantly different between follicular (1.9±0.7, 24.2±18.7) and luteal (1.9±0.3, 18.7±11.2) phases of menstrual cycle (P>0.05 respectively). In Symptomatic HIV females on HAART, there were no significant difference in the mean progesterone and estradiol concentrations between the follicular (2.5±2.0, 32.8±20.2) and luteal (2.1±1.8, 38.4±24.0) phases of menstrual cycle (P>0.05). But the mean serum progesterone and estradiol concentrations dropped significantly at follicular phase (4.4±2.5, 80.9±36.6) compared with the luteal phase (8.7±4.9, 94.1±50.1) of menstrual cycle in Control female subjects (P<0.05 respectively).

**Fig 2:**
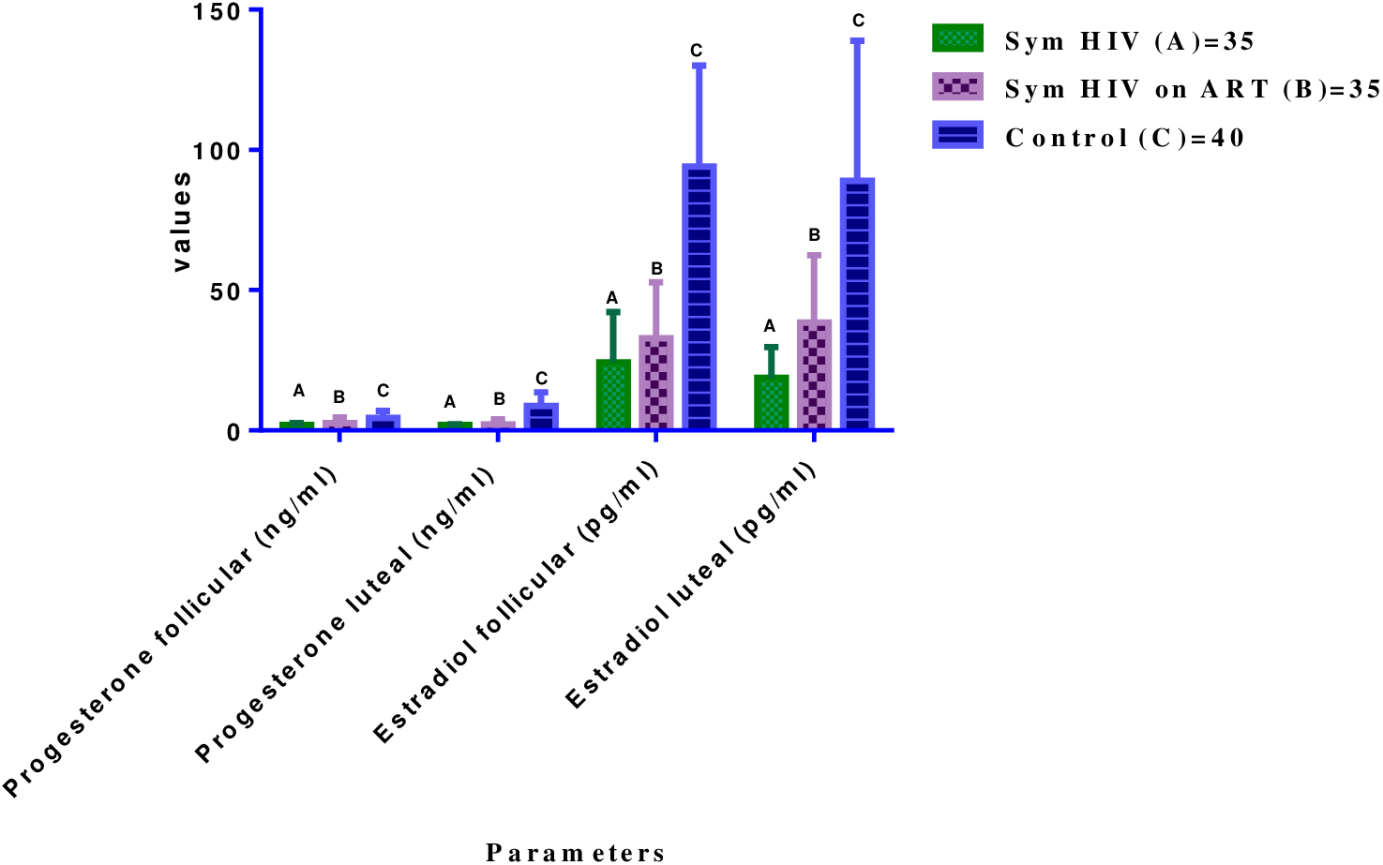
Comparison of mean (±SD) serum levels of progesterone and estradiol in test groups and control group at follicular and luteal phases of menstrual cycle. Key- Progesterone in all Test groups vs Control (C) = P<0.05 at both phases. Estradiol in all test groups vs control (C) = P<0.05 at follicular and luteal phases of menstrual cycle.

When the mean progesterone concentrations at follicular and luteal phases of menstrual cycle were compared between the Control group and the Test groups, the mean progesterone concentration dropped significantly in Symptomatic HIV (1.9±0.7, 24.2±18.3) and Symptomatic HIV on HAART (2.5±2.0, 32.8±20.2) compared with the corresponding values in the Control female subjects (4.4±2.5, 80.9±36.6) (P<0.05 respectively). Similarly, When the mean progesterone concentrations at luteal phase of menstrual cycle were compared between the Control group and the Test groups, the mean progesterone and estradiol values dropped significantly in Symptomatic HIV (1.9±0.3, 18.7±11.2) and Symptomatic HIV on HAART (2.1±1.8, 38.4±24.0) compared with corresponding values in the Control female subjects (8.7±4.9, 94.1±50.1) (P<0.05 respectively) (See Fig 2).

### Thyroid hormones {(ft3 (ng/ml) ft4 (μg/dl) and tsh (μiu/ml)} at follicular and luteal phases of menstrual cycle

The mean (±SD) serum FT3, FT4 and TSH levels in Symptomatic HIV females were not significantly different between follicular (0.73±0.37, 7.83±2.51, 4.17±3.65) and luteal (0.65±0.29, 8.71±2.20, 4.56±1.17) phases of menstrual cycle (P>0.05 respectively). There were no significant difference in the mean serum FT3 and FT4 concentrations (ng/ml) between follicular (1.11±0.30, 7.79±1.66) and luteal (0.95±0.31, 7.61±2.31) phases of the menstrual cycle in Symptomatic HIV females on HAART (P>0.05). On the contrary, the mean serum TSH value was significantly higher at follicular phase (3.26±1.88) compared with luteal phase (1.24±0.39) of menstrual cycle in Symptomatic HIV females on HAART (P<0.05). In Control female subjects, there were no significant difference in the mean serum FT3, FT4 and TSH concentrations (ng/ml) between follicular (1.01±0.48, 7.86±1.68, 1.32±0.49) and luteal (1.03±0.36, 7.11±2.03, 1.40±0.53) phases of menstrual cycle (P>0.05).

The mean FT3 at follicular and luteal phases dropped significantly in Symptomatic HIV females (0.73±0.37, 0.65±0.29) compared with the follicular and luteal values in Control female subjects (1.01±0.48, 1.03±0.36) (P<0.05 respectively). The post hoc analysis showed significant drop in the mean FT3 value (ng/ml) at follicular phase of menstrual cycle in Symptomatic HIV females (0.73±0.37, (0.65±0.29) compared with follicular value in the Symptomatic HIV females on HAART (1.11±0.30, 0.95±0.31 respectively) while the mean TSH was significantly higher in Symptomatic HIV females (4.17±3.65, 4.56±1.17) and Symptomatic HIV females on HAART (3.26±1.88, 4.81±3.96) at follicular and luteal phases compared with the corresponding values in the Control female subjects (1.32±0.49, 1.40±0.53) (P<0.05 respectively).

**Fig 3:**
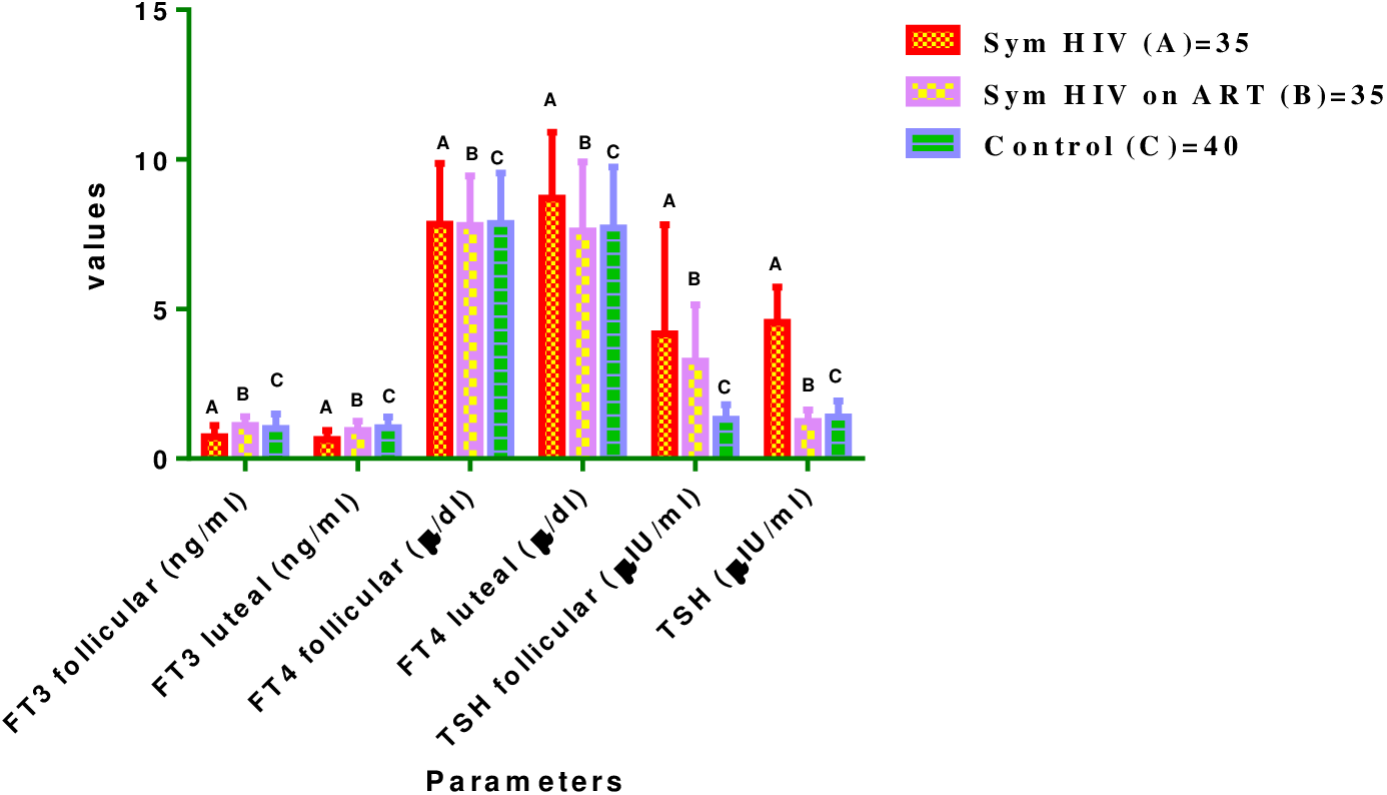
Comparison of mean (±SD) serum levels of FT3, FT4 and TSH in test groups and control group at follicular and luteal phases of menstrual cycle. Key—FT3 in A vs C = P <0.05 at both phases. A vs B = P < 0.05 at both phases of menstrual cycle. TSH in A vs C = P< 0.05 at both phases of menstrual cycle. B vs C = P< 0.05 at follicular phase. A vs B = P < 0.05 at luteal phase. TSH in B = P< 0.05 between follicular and luteal phases of menstrual cycle.

The post hoc analysis dropped significantly in the mean TSH value at luteal phase of menstrual cycle in Symptomatic HIV females on HAART (1.24±0.39) compared with Symptomatic HIV females (4.56±1.17) (P<0.05) (See Fig 3).

## Discussion

The present study showed that the mean serum levels of FSH, LH, progesterone and estradiol in Symptomatic HIV were not significantly different between follicular and luteal phases of menstrual cycle. This contrasts the observation in apparently healthy subjects where differences in hormonal levels exist between the two phases of the menstrual cycle. However, the significantly higher estradiol at luteal phase compared to follicular phase of menstrual cycle suggests some degree of impact of treatment on these subjects. FSH and LH are usually higher at the follicular phase and peak at the mid cycle to enable ovulation to take place [20] while Progesterone and estradiol are usually higher at the luteal phase. The absence of this normal physiological balance in HIV infected subjects may cause changes in the cycle which may affect reproductive function. Significant reductions in the level of progesterone at the luteal phase may disturb the sustenance of pregnancy and lead to spontaneous abortion while gross reductions in these hormones (hypogonadism) may cause failure of menstruation or significant abnormality in menstrual cycle [13]. However, Thyroid dysfunction has been reported to cause disturbances in the ovarian cycle and ovulation, but the molecular basis of the association is not known. It has been reported that Hypothyroidism causes decreased rates of metabolic clearance of androstenedione and estrone in women and unveils an increase in peripheral aromatization [21]. Hypothyroid women also exhibit decreased 5 α/β ratios of androgen metabolites, and also show an increase in excretion of 2-oxygenated estrogens [22]. In hypothyroidism, plasma binding activity of SHBG is decreased, which results in decreased plasma concentrations of both total testosterone and E2, but their unbound fractions are increased [23]. Altered metabolism of these gonadal steroids disappears when a euthyroid state is restored. The gonadotropins (Gn) level usually remains normal in hypothyroidism [24]. The present study was carried out in HIV individuals and the insignificant difference in the level of thyroid parameters (FT3, FT4 and TSH) observed in Symptomatic HIV, Symptomatic HIV females on HAART, between follicular and luteal phases of menstrual cycle suggests that the impact of drugs on thyroid function was not enough to return the subjects back to the pre disease states. However, the significantly lower level of FT3 with higher level of TSH observed in Symptomatic HIV females may suggest hypothyroidism possibly as result of HIV infection.

In normal healthy women, Ovarian follicles, from the pool of resting primordial follicles either continue to grow from preantral to antral follicles due to survival signals, such as gonadotrophins and growth factors, or degenerate and die by the process of follicular atresia. Expressions of hormones and growth factors have been shown to regulate the destiny of the ovarian follicle. In humans, disorders of the thyroid gland are responsible for a dysregulation of the hypothalamus, pituitary, gonadal axis, and hypothyroidism is associated with oligomenorrhea [10]. The follicular fluid composition might be the important regulator for developing oocytes and may play a substantial role in oocyte quality. Both FT3 and FT4 are found in the follicular fluid of humans, and a positive correlation was demonstrated between serum FT4 and follicular fluid FT4 levels [25]. The presence of thyroid hormone receptors in human oocytes may explain TH response on the ovaries. Both isoforms of TRs messenger RNA (mRNA) are expressed in the human oocyte. This showed that thyroid hormone may directly affect the oocyte [26] although; the presence of TRa and TRb mRNA in granulosa cells has been reported earlier [27]. Some authors have also demonstrated that TRa1 is expressed and localized in oocytes, granulosa cells and theca cells during follicular development [26, 27].

However, in the present study, the serum levels of FSH and LH were significantly higher while progesterone and estradiol levels were significantly lower in Symptomatic HIV, Symptomatic HIV on HAART at both follicular and luteal phases of menstrual cycle. The implication of grossly increased FSH with reduced progesterone is primary ovarian failure. This means that the anterior pituitary is overworking itself to stimulate a poorly responding ovary. The abnormally low progesterone signifies hypogonadism and may lead to menstrual and reproductive failure [13]. Normally, in naïve HIV women, serum level of FSH and LH are significantly low in cases of overt hypothyroidism when done between day 2 and 5 of the cycle [28]. Studies have also demonstrated that serum estradiol was reduced significantly in the hypothyroid state when compared to the control [29] while Hapon *et al*. reported that hypothyroidism does not influence the classical preovulatory patterns of LH and FSH secretion in rats [30].

The thyroid hormone plays a vital role in all physiological activities in humans including menstrual functions in females. Increased thyroid function (hyperthyroidism) may lead to premature menstruation or precotious puberty, menorrhagia or hypermenorrhoea whereas reduced thyroid function (hypothyroidism) may lead to delayed menstruation or oligomenorrhoea and pregnancy loss [31, 32]. This has been attributed to the connection between thyroid hormone levels and the menstrual cycle which is mainly mediated by thyrotropin-releasing hormone (TRH), which has a direct effect on the ovary. Additionally, abnormal thyroid function can alter levels of sex hormone-binding globulin, prolactin, and gonadotropin-releasing hormone, contributing to menstrual dysfunction. For example, increased levels of TRH may raise prolactin levels, contributing to the amenorrhea associated with hypothyroidism [5].

Further analysis showed that seventeen out of twenty (85%) Symptomatic HIV female subjects had significantly higher FSH and LH levels while progesterone and estradiol were being secreted below the lower limit of normal. This strongly indicates a state of reduced reproductive hormones causing hypogonadism. The significantly reduced levels of progesterone and estradiol (the two major female sex hormones) probably as a result of HIV infections [4] send a positive feedback to the anterior pituitary glands causing them to over secrete FSH and LH [13]. Some studies done in HIV subjects in the developed countries produced a similar report [33, 34]. However, it has been recently reported that hypothyroidism diminished serum E2 concentrations in women at their reproductive age group [35].

The reduction of FSH, appreciation in the level of progesterone with the insignificant difference in the levels of FT3 and TSH observed in HIV subjects on HAART suggests stimulatory effects of the treatment on the gonads and possible reduction on the incidence of hypothyroidism with intact negative feedback mechanism thereby resulting in the restoration of the gonadal functions showing some beneficial effects and a tendency to return to normal. This may reduce the incidence of menstrual abnormality and infertility. Studies have demonstrated a positive correlation between TSH and PRL in hypothyroid women [36]. In a previous study, it was shown that FT4 administration in hypothyroidism normalizes PRL and LH levels, increased folliculogenesis and estradiol secretion, reverses menstrual abnormalities and increases spontaneous fertility [37]. FT3 is considered a biological amplifier of the stimulatory action of gonadotrophins on granulosa cell function [35].

HIV disease is associated with opportunistic infections which are linked with increased levels of pro- inflammatory cytokines. The later may be responsible for the hypogonadism observed in HIV, which calls for further investigations. However, prolonged use of HAART (protease inhibitors) has been associated with thyroid abnormalities such as Hashimoto’s thyroiditis which results to a kind of immune reconstitution [38–40]. However, the present study was carried out in women within their reproductive age at different phases of menstrual cycle and did not observe this drug effects.

The present study did not find any significant association between age and thyroid hormones nor reproductive hormones assayed in the affected women. Thyroid hormones were not significantly associated with viral load, nadir CD4+ count and duration of HAART. Previous report has shown that FT3 and FT4 were related to the state of HIV infection and are potential biomarkers of HIV progression [41]. Beltral *et al* found that only stavudine treatment and low CD4 count were statistically associated with hypothyroidism [15] while Madedu *et al*. found that TSH levels were negatively correlated with CD4+ count nadir [16] which was consistent with the findings in the present study. The present study also observed that FT3, FT4, Prog and E2 were inversely correlated while FSH and LH were positively correlated with duration of HIV infection in HIV females. This was similar to the previous reports by Roksana *et al*., [42] and Shujinj *et al*., [41]). It is still very unclear if the cause of thyroid dysfunction in HIV patients is the HIV infection itself, its complication, therapy or progression [16, 43, 44] as thyroid dysfunction has been reported before the introduction of HAART which was the case with the present study. However, the present study observed hypothyroidism in symptomatic HIV infected females who were not on HAART. Bongiovanni et al. reported that therapy had an acute influence on thyroid function [45] while Silva *et al*. suggested that immune reconstitution was more likely to protect the thyroid function than impair it [46].

The present study reported that the percentage of menstrual irregularities was higher in symptomatic HIV infected females 18(40%) as compared to symptomatic HIV infected females on HAART 28(25.7%). This was supported by the significant hypogonadism reported in the present study. Dobs *et al*. in their similar study reported hypogonadism in 6%, 40%, and 50% of asymptomatic HIV positive, symptomatic HIV positive and AIDS patients respectively [47]. Meena *et al*. reported that primary hypogonadism was more common cause of hypogonadism [48, 49] while secondary hypogonadism was due to decrease in gonadotropin secretion during acute or chronic severe illness and involvement of hypothalamic or pituitary tissue by opportunistic infections or malignancies in both sexes [50, 51]. Primary gonadal failure was attributed to opportunistic infections such as (Cytomegalo virus, Mycobacterium avium complex, Cryptococcus neoformans etc) infiltration by a neoplasm like Kaposi’s sarcoma, IL 1 and tumor necrosis factor (TNF) that decreases the leydig cell steroidogenesis [52, 53].

In conclusion, the present study demonstrated hypothyroidism with significant degree of primary hypogonadism in Symptomatic HIV infected females at both follicular and luteal phases of menstrual cycle which tends to normalize on treatment. A counselling module for reproduction and routine screening for thyroid function is therefore advocated to be included in voluntary counselling of HIV subjects to reduce the incidence of reproductive and thyroid dysfunctions in affected women.

## Supporting information

S1 Questionnaire (DOC)
