# Supplementary material for: Impact of thyroid function abnormalities on reproductive hormones during menstrual cycle in premenopausal HIV infected females at NAUTH, Nnewi, Nigeria
Source: PLoS One. 2017 Jul 19;12(7):e0176361. doi: 10.1371/journal.pone.0176361 (PMC5516981; doi:10.1371/journal.pone.0176361)
Supplement: S1 Questionnaire — (DOC) [file pone.0176361.s001.doc]

**APPENDIX**

**QUESTIONNAIRE**

The following question requires voluntary and truthful response. The information given is considered highly confidential.

# Section A: BIODATA

Code no:

Age

Sex: a) female b) male

Occupation:

Religion: a) Christian b) Moslem c) Traditional worshiper

Level of education: a) primary b) secondary c) tertiary

Marital status: a) married b) single c) separated

# SECTION B: DURATION OF ILLNESS

1. When did you test positive for HIV infection?

a) A month ago; b) 6 months ago; c) 12 months ago d) >18 months

# SECTION C MENSTRUAL HISTORY

1. Do you see your menses? Yes/No
2. When last did you see your menses?
3. How many days do your menses flow?

(a 1 day, (b 2days, (c 3days, (d 4days, (e 5days, (e 6days, (f more than 7days.

1. Have you experienced any problem with your menses? Yes/No
2. What type or nature of problem?
   1. Excess flow b) too little flow c) painful period d) cessation of menses e) too frequent menses f) any other problem. (Specify)
3. Are you pregnant? Yes/No
4. Have been diagnosed of any thyroid disease? Yes/No
5. If yes, have you been on treatment? Yes/No
6. Are you on any contraceptives? Yes/No
7. If yes, what type? And for how long?
8. Do you smoke? Yes/No

**SECTION D ANTIRETROVIRAL HISTORY**

11. Are you on drug (ART)? Yes/No

12. How long have you been on ART?

1. 1-3months b) 4-6months c) 6 -12months d) >12 months

13. When did your menstrual problem start?

a) Before I started ART b) After I started ART

14. Is the problem better after taking your drugs?

a) Has improved b) No change c) Has got worse

**SECTION E TUBERCULOSIS**

15. Have you been tested for TB? Yes/No

16. When did you test positive?

a) A month ago; b) 6 months ago; c) 12 months ago d) >18 months ago

17. Have you been placed on Anti TB drug? Yes/No

18. How long have you been on drugs?

1. 1-3months b) 4-6months c) 6 -12months d) >12 months

**SECTION F: HEPATITIS**

19. Have you been tested for Hepatitis B and C? Yes/No

20. If yes, when did you test positive?

a) A month ago; b) 6 months ago; c) 12 months ago d) >18 months ago

21. Have you been on treatment? Yes/No
